# Perioperative Complete Blood Count Changes After Endovascular Aneurysm Repair (EVAR) and Fenestrated EVAR (FEVAR): A Retrospective Cohort Study

**DOI:** 10.7759/cureus.105733

**Published:** 2026-03-23

**Authors:** Yusuke Iizuka, Takuya Kosaka, Takahiro Kishi, Hiroshi Mitsuoka, Yuki Yamaguchi, Akihiro Hoshino, Hidemichi Masuda

**Affiliations:** 1 Department of Radiation Oncology, Shizuoka City Shizuoka Hospital, Shizuoka, JPN; 2 Department of Radiation Oncology and Image-Applied Therapy, Graduate School of Medicine, Kyoto University, Kyoto, JPN; 3 Department of Cardiovascular Surgery, Shizuoka City Shizuoka Hospital, Shizuoka, JPN; 4 Department of Radiation Technology, Shizuoka City Shizuoka Hospital, Shizuoka, JPN

**Keywords:** aortic aneurysm stent grafting, endovascular aneurysm repair, fenestrated endovascular aneurysm repair, interventional radiation exposure, perioperative blood count

## Abstract

Background

Endovascular aneurysm repair (EVAR) and fenestrated EVAR (FEVAR) expose the abdominal and pelvic regions to localized radiation, but their hematologic effects remain unclear.

Purpose

To characterize perioperative changes in complete blood count (CBC) after EVAR/FEVAR and to explore potential associations with radiation exposure in a hypothesis-generating manner.

Methods

We retrospectively analyzed 75 procedures (64 EVAR, 11 FEVAR) performed in 2022. Hemoglobin (Hb), white blood cells (WBC), neutrophils (Neu), lymphocytes (Lym), and platelets (Plt) were measured at baseline and on postoperative day 1, and at one week, one month, and six months after intervention. Associations with radiation dose (reference-point air kerma), blood loss, and patient characteristics were evaluated.

Results

CBC parameters showed characteristic early postoperative fluctuations on postoperative day 1: Hb and Plt decreased, Lym declined markedly, and WBC and Neu increased (all P < 0.01). Most values approached baseline by six months. No clear dose-response relationship was observed in the overall cohort; however, exploratory subgroup analyses suggested more sustained reductions in Hb and Lym among males, patients with BMI <23 kg/m², and those receiving ≥1 Gy.

Conclusion

EVAR/FEVAR were associated with transient perioperative CBC shifts that largely resolved within six months. While radiation dose was not a clear determinant of hematologic changes in the overall cohort, exploratory subgroup findings suggested possible differences in patients with higher exposure or lower BMI; these observations should be interpreted as hypothesis-generating. Further investigations incorporating organ-specific dosimetry are needed to better understand potential hematopoietic effects of complex endovascular procedures.

## Introduction

Endovascular aneurysm repair (EVAR) and fenestrated EVAR (FEVAR) are widely used minimally invasive treatments for abdominal and complex aortic aneurysms. Compared with open repair, these procedures offer reduced perioperative morbidity and faster recovery, contributing to their increasing adoption in aging populations [1-3]. FEVAR, in particular, often requires prolonged and complex fluoroscopic guidance to achieve precise alignment of fenestrations with target branch vessels [4,5], resulting in increased radiation exposure to the abdomen and pelvis. In clinical practice, radiation exposure during EVAR/FEVAR is commonly assessed using reference-point air kerma (Ka,r), which, although not a direct measure of organ dose, serves as a practical surrogate for relative exposure. Previous studies have reported that Ka,r during EVAR typically ranges around 1-3 Gy and can be substantially higher in complex procedures such as FEVAR.

While the dermatologic effects of high-dose interventional procedures are well established [6], the hematologic consequences of localized fluoroscopic exposure remain less well characterized. This represents an important knowledge gap, as a substantial proportion of active adult bone marrow is located within the lumbar spine, pelvis, and proximal femora, regions directly exposed during EVAR/FEVAR [7]. Moreover, recent dosimetric and simulation studies suggest that even low-energy X-ray beams used in interventional procedures can contribute measurable marrow doses [8].

Perioperative complete blood count (CBC) changes following major surgery are common and reflect inflammatory stress, hemodilution, and blood loss. However, lymphocytes are among the most radiosensitive cell populations and may undergo apoptosis even at relatively low absorbed doses [9,10]. It is therefore biologically plausible that radiation exposure during prolonged EVAR/FEVAR procedures could influence postoperative CBC dynamics, particularly lymphocyte counts. Despite this rationale, clinical evidence remains limited, and it is unclear whether perioperative lymphopenia extends beyond the expected postoperative stress response or is associated with radiation exposure during EVAR/FEVAR.

Therefore, the aim of this study was to characterize perioperative CBC changes following EVAR and FEVAR and to explore their association with procedural factors, including radiation exposure, in a hypothesis-generating manner. We hypothesized that higher radiation exposure during EVAR/FEVAR would be associated with greater early lymphocyte decline and/or delayed hematologic recovery.

## Materials and methods

Study design and population

This retrospective observational study was conducted at Shizuoka City Shizuoka Hospital and approved by the institutional review board (23-01). All procedures adhered to the principles of the Declaration of Helsinki. Informed consent was obtained through an opt-out process posted on the hospital website. All patients who underwent EVAR or FEVAR between January and December 2022 were screened, and all eligible procedures were consecutively included. No patients were excluded because of missing baseline laboratory data. Of 76 procedures screened, 75 were included in the final analysis after exclusion of one patient with idiopathic thrombocytopenic purpura who required perioperative platelet transfusion.

For patients undergoing multiple staged procedures, follow-up for the first procedure was censored at the time of the second procedure to avoid overlapping observation periods, and the latter was treated as a separate case in the analysis. Patients whose follow-up blood tests were unavailable or who developed conditions affecting hematologic parameters (e.g., chemotherapy, severe infection) during follow-up were excluded from analyses beyond the affected time points.

Study procedures

EVAR procedures were performed using the Azurion 7 B20/15 robotic C-arm system (Philips Healthcare, Best, Netherlands) or the Artis Zeego system (Siemens Healthcare, Erlangen, Germany). All FEVAR procedures were performed using the Artis Zeego system. Radiation exposure was monitored using reference-point air kerma (Ka,r), automatically recorded by the fluoroscopy system. Although air kerma does not directly represent organ-absorbed marrow dose, it is routinely used as a standard dosimetric index in interventional radiology and provides a practical surrogate for relative exposure comparisons in abdominal procedures. Additional radiation metrics such as fluoroscopy time, dose-area product, or beam angulation were not systematically available in this retrospective dataset.

Data collection

CBC parameters, including hemoglobin (Hb), white blood cells (WBC), neutrophils (Neu), lymphocytes (Lym), and platelets (Plt), were measured at the following clinically defined time points: within one week before the procedure (baseline), postoperative day 1, approximately one week, approximately one month, and three months or later. Follow-up blood tests were performed according to routine clinical practice rather than a predefined study protocol. Because the timing of postoperative blood tests varied among patients, median sampling days were used to define the week-1, month-1, and six-month time points (median six days {interquartile range (IQR) 5-7}, 30 days {IQR 25-33}, and 180 days {IQR 119-188}, respectively), with the ≥3-month category corresponding to the six-month time point for analysis. All tests were performed using the Sysmex XR-3000 automated hematology analyzer (Sysmex Corporation, Kobe, Japan).

Demographic information (age, sex, height, weight, and BMI), procedure type (EVAR or FEVAR), intraoperative factors (blood loss, transfusion volume, and radiation dose), and postoperative complications, including any skin findings suggestive of radiation injury, were extracted from the medical records. When applicable, net blood loss was calculated as the difference between intraoperative blood loss and transfusion volume.

Endpoints

The primary endpoint was the magnitude and trajectory of perioperative CBC changes, particularly at the six-month time point. Secondary endpoints included associations between CBC changes and procedural factors (including radiation dose), the effects of blood loss or transfusion, and subgroup differences based on sex, age, BMI, and radiation exposure level.

Statistical analysis

Continuous variables are presented as means ± standard deviations or medians with ranges, as appropriate. The distribution of continuous variables was assessed using the Shapiro-Wilk test. CBC parameters at each postoperative time point were compared with baseline values using paired t-tests. Subgroup comparisons were performed using paired t-tests within groups and independent t-tests between groups. Associations with blood loss variables were evaluated using Pearson's correlation coefficients, whereas radiation dose was assessed using Spearman's rank correlation coefficients. Two-sided P values < 0.05 were considered statistically significant. All analyses were conducted using R version 4.4.1 (R Foundation for Statistical Computing, Vienna, Austria). No proprietary scales, copyrighted scoring systems, or licensed assessment tools were used in this study. Given the exploratory nature of the study, analyses were primarily based on paired comparisons relative to baseline, and adjustments for multiple comparisons were not applied.

## Results

Patients’ characteristics

A total of 75 procedures were included in the final analysis. The median age was 76 years (range, 48-98), and 60 patients (80%) were male. The median reference-point air kerma was 1.00 Gy (range, 0.15-25.70 Gy). The highest value (25.7 Gy) occurred in a complex FEVAR procedure and was confirmed by review of the procedural radiation record. Seven procedures exceeded 5 Gy of reference-point air kerma. The mean intraoperative blood loss was 150 mL (range, 0-1,053 mL), and 16 patients received transfusions (mean 690 mL). No cases of radiation dermatitis were documented in the medical records during hospitalization or follow-up visits.

Perioperative changes in CBC

CBC measurements were available for 75 patients on postoperative day 1, 72 at approximately one week, 63 at approximately one month, and 48 at six months (median 180 days). Loss to follow-up at six months was primarily due to variation in routine clinical follow-up schedules rather than clinical deterioration. Significant postoperative shifts were observed across all CBC parameters on postoperative day 1 compared with baseline (all P < 0.01). Hb decreased from 12.8 ± 1.8 to 10.9 ± 1.4 g/dL, Lym from 1.55 ± 0.51 to 0.90 ± 0.35 ×10³/µL, and Plt from 18.8 ± 6.1 to 14.7 ± 5.0 ×10⁴/µL, whereas WBC and Neu increased.

**Figure 1:**
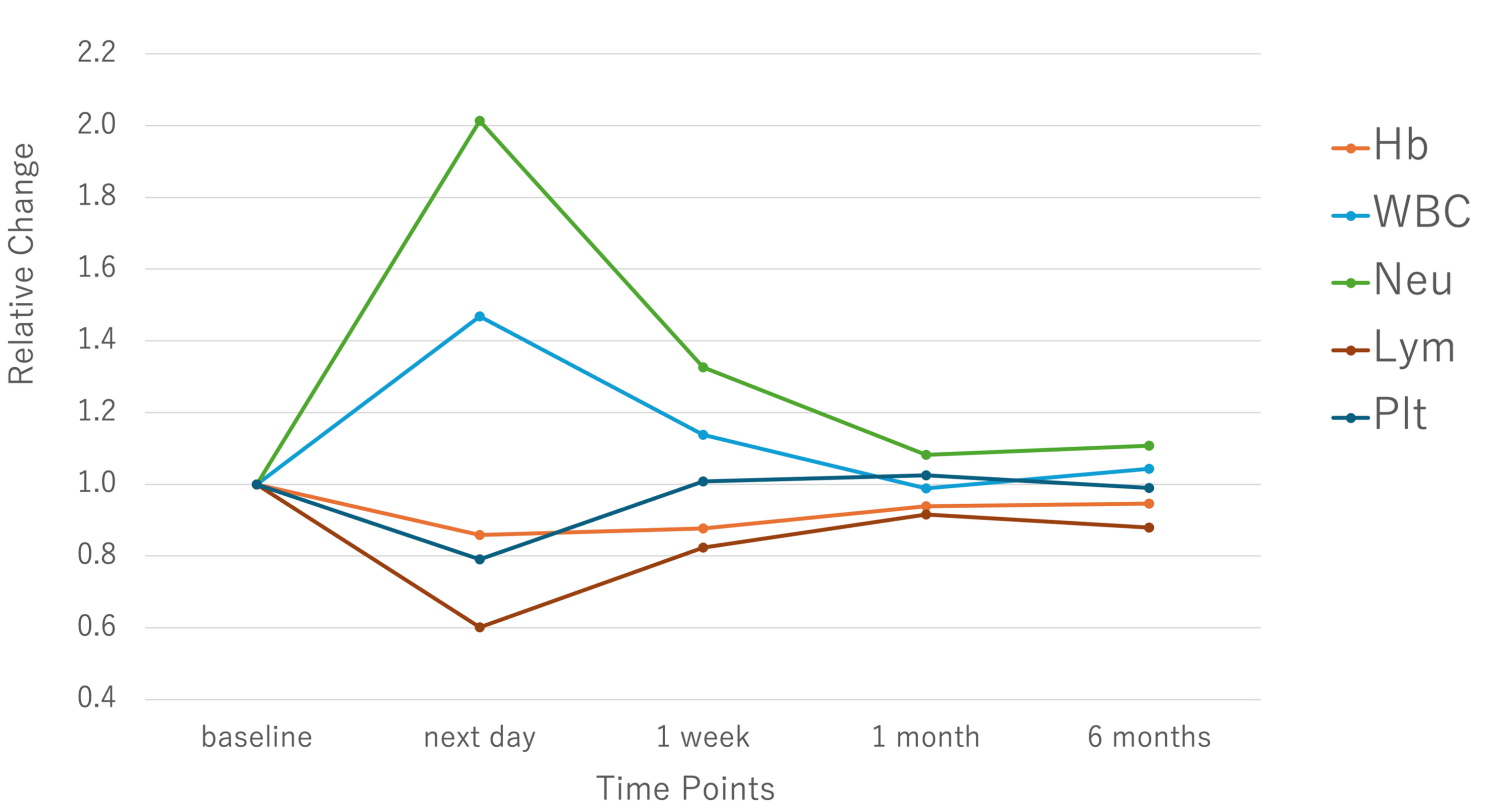
Relative change in complete blood count parameters over time after endovascular aneurysm repair or fenestrated endovascular aneurysm repair. Changes in complete blood counts are expressed relative to the baseline values across various time points. Hb, hemoglobin; WBC, white blood cell count; Neu, neutrophil count; Lym, lymphocyte count; Plt, platelet count.

These early changes gradually improved over the subsequent weeks. By one month, most parameters approached baseline, and by six months, only Hb remained slightly but significantly lower than preoperative levels (12.1 ± 2.0 vs. 12.8 ± 1.8 g/dL; P = 0.02). Longitudinal trends are shown in Table 1 and Figure 1.

**Table 1:** Complete blood count changes in all patients. The ratios in parentheses represent the average ratio of values compared to baseline. * P < .05 compared to baseline. EVAR, endovascular aneurysm repair; Hb, hemoglobin; WBC, white blood cell count; Neu, neutrophil count; Lym, lymphocyte count; Plt, platelet count.

Parameter	Baseline (n=75)	1 day post-EVAR (n=75)	1 week post-EVAR (n=72)	1 month post-EVAR (n=63)	6 months post-EVAR (n=48)
Hb (g/dL)	12.8±1.8	10.9±1.4* (0.86*)	11.1±1.6* (0.88*)	12.0±1.6 (0.94*)	12.1±2.0* (0.95*)
WBC (x10^3^/μL)	6.19±1.99	8.76±2.83* (1.47*)	6.83±2.36 (1.14*)	5.91±2.04 (0.99)	6.12±1.87 (1.04)
Neu (x10^3^/μL)	4.01±1.84	7.08±2.73* (2.01*)	4.76±2.23* (1.33*)	3.92±1.97 (1.08)	3.84±1.64 (1.11)
Lym (x10^3^/μL)	1.55±0.51	0.90±0.35* (0.60*)	1.22±0.44* (0.82*)	1.45±0.56 (0.92*)	1.43±0.57 (0.88*)
Plt (x10^4^/μL)	18.8±6.1	14.7±5.0* (0.79*)	18.8±7.5 (1.01)	18.7±6.3 (1.03)	18.0±7.4 (0.99)

Relationship with blood loss and transfusion

A weak correlation was observed between postoperative Hb change and intraoperative blood loss when assessed as an absolute value (r = 0.29, P = 0.011), whereas no significant correlation was observed when assessed as a ratio (r = 0.17, P = 0.15). Adjustment for transfusion volume did not alter the results, and the association with net blood loss remained weak (r = −0.28, P = 0.015).

Subgroup analyses

Patients were stratified by sex, age, BMI, and radiation dose using median cutoffs (Table 2).

**Table 2:** Complete blood count at baseline and six months after EVAR in different groups. The ratios in parentheses represent the average ratio of values compared to baseline. * P < .05 between baseline and 6 months post-EVAR within the subgroups. † P < .05 between the subgroups. EVAR, endovascular aneurysm repair; Hb, hemoglobin; WBC, white blood cell count; Neu, neutrophil count; Lym, lymphocyte count; Plt, platelet count. Reference ranges at our institution were as follows: hemoglobin (Hb): 13.5–17.0 g/dL (male) and 11.5–15.0 g/dL (female); white blood cell count (WBC): 3.5–9.0 ×10³/µL; neutrophils (Neu): 1.5–7.5 ×10³/µL; lymphocytes (Lym): 1.0–3.5 ×10³/µL; and platelets (Plt): 15–35 ×10⁴/µL.

Subgroup	Category	Hb (g/dL)	WBC (x10^3^/μL)	Neu (x10^3^/μL)	Lym (x10^3^/μL)	Plt (x10^4^/μL)
Sex						
Male (n=64)	Baseline	13.0±1.7	6.26±2.06	4.05±1.94	1.56±0.52	19.2±6.1
	6 months	12.1±2.1* (0.94*)	6.32±1.90 (1.06)	3.99±1.67 (1.15*)	1.45±0.56 (0.87*)	18.8±7.5 (0.99)
Female (n=11)	Baseline	11.9±1.6	5.78±1.50	3.76±1.17	1.51±0.42	16.4±5.6
	6 months	11.2±1.1 (0.95)	5.39±1.23 (0.95)	3.57±1.16 (0.97)	1.28±0.38 (0.89)	15.5±4.4 (1.09)
Age (years)						
<76 (n=35)	Baseline	13.1±1.8	6.09±1.56	3.81±1.38	1.63±0.47	20.2±6.8
	6 months	12.3±1.8 (0.92)	6.30±2.00 (1.04)	3.88±1.86 (1.24)	1.39±0.51 (0.83)	19.8±6.4 (0.98)
≥76 (n=40)	Baseline	12.6±1.8	6.28±2.32	4.18±2.17	1.49±0.54	17.5±5.2
	6 months	12.0±2.1 (0.97)	5.99±1.79 (1.04)	3.81±1.54 (1.02)	1.47±0.67 (0.91)	16.7±7.9 (1.00)
BMI (kg/m^2^)						
< 23 (n=36)	Baseline	12.3±1.6†	6.04±1.86	3.88±1.72	1.52±0.52	18.7±6.0
	6 months	11.4±1.8* (0.95*)	6.31±2.21 (1.10*)	4.05±2.06 (1.16)	1.46±0.52 (0.87*)	17.6±8.1 (1.02)
≥ 23 (n=39)	Baseline	13.3±1.8†	6.33±2.12	4.13±1.96	1.58±0.51	18.8±6.3
	6 months	12.9±1.9 (0.94)	5.91±1.43 (0.98)	3.59±1.01 (1.05)	1.41±0.68 (0.89)	18.3±6.7 (0.96)
Radiation dose (Gy)						
< 1.0 (n=38)	Baseline	12.3±1.6†	6.30±2.31	4.22±2.22	1.45±0.47	20.0±7.0
	6 months	11.8±2.1 (0.96*)	6.18±3.30 (1.08)	3.90±1.66 (1.10)	1.56±0.63 (0.93)	18.7±9.4 (0.96)
≥ 1.0 (n=37)	Baseline	13.4±1.7†	6.09±1.61	3.79±1.38	1.65±0.53	17.5±4.8
	6 months	12.4±1.9* (0.93*)	6.07±1.92 (1.01)	3.78±1.67 (1.11)	1.32±0.49* (0.84*)	17.2±4.6 (1.02)

Although within-group decreases were observed, between-group comparisons did not reach statistical significance. Specifically, (i) male patients showed persistent reductions in Hb (P=0.02), Neu (P=0.03), and Lym (P<0.01) at six months; (ii) patients with BMI < 23 kg/m2 demonstrated significant decreases in Hb and Lym at 6 months (both P<0.02); and (iii) patients receiving ≥ 1.0 Gy exhibited significant 6-month declines in Hb (P<0.01) and Lym (P<0.01), whereas those receiving < 1.0 Gy also showed a modest, but significant Hb decrease (P=0.04).

These findings suggest that although the overall cohort did not show clear dose-response relationships, specific subgroups, particularly those with higher radiation exposure or lower BMI, may suggest differences in hematologic recovery; however, these findings should be interpreted cautiously and considered exploratory.

## Discussion

This study evaluated perioperative hematologic changes following EVAR and FEVAR and explored their potential association with procedural radiation exposure. The major findings were as follows: (1) CBC parameters demonstrated characteristic early postoperative fluctuations, including transient decreases in Hb, Lym, and Plt and increases in WBC and Neu; (ii) most values returned to near-baseline levels by six months; and (iii) although no clear dose-response relationships were identified in the overall cohort, certain subgroups, particularly patients with higher radiation exposure or lower BMI suggested more sustained reductions in Hb and Lym. These results suggest that perioperative hematologic changes after EVAR/FEVAR are largely attributable to surgical and inflammatory factors, while radiation exposure could theoretically contribute; however, our analysis did not demonstrate a clear dose-response relationship.

The early postoperative rise in WBC and Neu and the decrease in Lym are consistent with the well-described systemic inflammatory response induced by endovascular manipulation. The magnitude of these early changes was clinically notable, with hemoglobin decreasing by approximately 1.9 g/dL and lymphocyte counts declining by about 40% on postoperative day 1 compared with baseline. Surgical stress, hemodilution, blood loss, and perioperative fluid shifts are known contributors to these changes. Perioperative fluid administration may further contribute to early postoperative hemoglobin decline through hemodilution; however, detailed data on crystalloid or colloid volumes were not consistently available in this retrospective dataset [11]. The prominent early lymphopenia observed in our cohort may also reflect the radiosensitivity of lymphocytes, which undergo apoptosis even after relatively low absorbed doses [9,10]. Although fluoroscopic radiation during EVAR is localized, substantial irradiation of marrow-rich pelvic and lumbar structures is possible, particularly in complex procedures such as FEVAR. In our cohort, the highest reference-point air kerma reached 25.7 Gy in a complex FEVAR procedure, illustrating the potential for substantial radiation exposure during technically demanding interventions. Simulation-based studies have demonstrated that even low-energy X-rays used in interventional radiology can contribute measurable bone marrow doses [8].

Subgroup analyses suggested potential heterogeneity in hematologic response. Patients receiving ≥1.0 Gy of reference-point air kerma demonstrated more persistent reductions in Hb and Lym. Although air kerma is an imperfect surrogate for marrow dose, it correlates with procedural complexity, fluoroscopy time, and irradiated volume, and therefore serves as a practical index of relative radiation exposure in abdominal interventions. Similarly, patients with lower BMI may absorb less scatter and attenuate less of the beam, potentially resulting in proportionally higher organ doses. Differences in body habitus may therefore influence radiation distribution and attenuation during fluoroscopic procedures. However, although within-group decreases were observed, between-group comparisons did not reach statistical significance, and these findings should be interpreted cautiously as exploratory rather than evidence of a true subgroup effect.

Comparisons with radiation-related hematologic effects in other clinical settings are informative. Radioiodine therapy, for example, produces dose-dependent lymphopenia and transient reductions in WBC and Plt counts [12-14]. Although radionuclide therapy delivers substantially higher systemic doses than fluoroscopic imaging, both modalities share the principle that lymphocytes are among the first cell populations to be affected. In our study, the magnitude of lymphocyte reduction was smaller, and recovery was faster, consistent with the localized and relatively lower dose delivered during EVAR/FEVAR. Nonetheless, the persistence of decreased Lym and Hb in selected subgroups suggests that the hematologic impact of complex endovascular procedures warrants further investigation.

From a clinical perspective, hematologic changes after EVAR are usually considered benign; however, our findings suggest that mild delayed suppression may occur in specific patients, such as those undergoing high-dose or prolonged procedures. Although the clinical consequences of these changes are uncertain, awareness of potential lymphopenia may be relevant in vulnerable populations. No clinically significant cytopenia requiring intervention was observed in our cohort. As EVAR and FEVAR techniques become increasingly complex and fluoroscopic times lengthen, understanding the cumulative hematologic impact may become increasingly important [15].

The observed perioperative hematologic changes are likely multifactorial and predominantly driven by surgical stress, hemodilution, perioperative fluid shifts, and blood loss. Associations between radiation indices and CBC changes should therefore be interpreted as hypothesis-generating, as causal inference is limited by the retrospective design and the use of air kerma rather than organ-specific marrow dose. These findings did not alter perioperative management in our cohort.

This study has several limitations. First, its single-center retrospective design with a modest sample size and a limited number of FEVAR procedures may restrict generalizability and preclude procedure-specific analyses. Second, reference-point air kerma is a surrogate for radiation exposure and does not directly represent absorbed bone marrow dose; more precise dosimetric approaches would be required for definitive dose-effect evaluation. Third, important perioperative variables-including contrast volume, procedure duration, inflammatory markers, and additional radiation metrics-were not consistently available, and unmeasured confounding factors such as perioperative medications, infection, and fluid administration may have influenced hematologic trends. Fourth, the timing of postoperative blood sampling varied, and incomplete follow-up at six months may introduce bias, although this likely reflects variability in routine clinical practice rather than patient condition. Finally, repeated measures, multiple comparisons, and the absence of longitudinal modeling approaches limit statistical inference, and all findings should be interpreted as exploratory and hypothesis-generating.

## Conclusions

EVAR/FEVAR were associated with transient perioperative CBC shifts that largely resolved within six months. While radiation dose was not an independent determinant in the overall cohort, exploratory subgroup analyses suggested possible differences in patients with higher radiation exposure or lower BMI, but these findings should be interpreted as hypothesis-generating and require confirmation in larger studies. Further investigations incorporating organ-specific dosimetry are needed to better understand potential hematopoietic effects of complex endovascular procedures.
